# CD40 Accelerates the Antigen-Specific Stem-Like Memory CD8^+^ T Cells Formation and Human Papilloma Virus (HPV)-Positive Tumor Eradication

**DOI:** 10.3389/fimmu.2020.01012

**Published:** 2020-05-27

**Authors:** Yanmei Zhang, Nisha Wang, Meilin Ding, Yang Yang, Zhimin Wang, Lei Huang, Wei Zhu, Andrew L. Mellor, Xiaorui Hou, Chenfei Zhou, Ruiming Yan, Wei Wang, Sha Wu

**Affiliations:** ^1^Department of Immunology, School of Basic Medical Sciences, Southern Medical University, Guangdong Provincial Key Laboratory of Proteomics, Guangzhou, China; ^2^Guangdong Provincial Key Laboratory of Single Cell Technology and Application, Department of Biochemistry and Molecular Biology, School of Basic Medical Sciences, Southern Medical University, Guangzhou, China; ^3^Department of Obstetrics and Gynecology, The First Affiliated Hospital of Guangzhou Medical University, Guangzhou, China; ^4^Department of Obstetrics and Gynecology, The Six Affiliated Hospital of Guangzhou Medical University, Qingyuan, China; ^5^Center for Gene and Cellular Immunotherapy, National Center for the International Research in Cell and Gene Therapy, School of Basic Medical Sciences, Academy of Medical Sciences, Zhengzhou University, Zhengzhou, China; ^6^Faculty of Medical Sciences, Framlington Place, Institute of Cellular Medicine, Newcastle University, Newcastle-Upon-Tyne, United Kingdom; ^7^Hepatology Unit and Department of Infectious Diseases, Nanfang Hospital, Southern Medical University, Guangzhou, China; ^8^Department of Obstetrics and Gynecology, Guangdong Provincial People's Hospital, Guangdong Academy of Medical Sciences, Guangzhou, China

**Keywords:** Tscm, CD40, HPV, Wnt/β-catenin, cancer

## Abstract

Antigen-specific stem-like memory CD8^+^ T cells (Tscm) have a series of stem cell characteristics, including long-term survival, self-renewal, anti-apoptosis and persistent differentiation into cytotoxic T cells. The effective induction of tumor-specific CD8^+^ Tscm could persistently eradicate tumor in pro-tumor hostile microenvironment. This study was to investigate the role of CD40 in HPV16-specific CD8^+^ Tscm induction and its anti-tumor function. We found that CD40 activation accelerated vaccine-induced HPV16 E7-specific CD8^+^ Tscm formation. Comparing to other HPV-specific CD8^+^ T cells, CD8^+^ Tscm were found to be stronger and long-term anti-tumor function, *in vivo* and *in vitro*, even in the adoptive cellular transferring model. Furthermore, high frequencies of Tscm might prevent the HPV infection to move on to the development of cancer. And the CD40 effect on Tscm involved Wnt/β-catenin activation. Our study suggest that CD40 activation supports the generation of tumor-specific CD8^+^ Tscm, thus providing new insight into cancer immunotherapy.

## Introduction

Approximately 57,000 new cases of cervical cancer all over the world take place in 2018, making it the 2nd most common female cancer and the 4th fatal female malignancy worldwide (1). HPV vaccines are proved to be preventive from human papillomavirus infection and related cervical cancer, but ineffective to the established cervical cancer (2). The HPV-specific cytotoxic T cells dominate anti-tumor immunity against cervical cancer. Recently, multiple CD8^+^ T-based adoptive cells therapies (ACT), including TIL, CAR-T and TCR-T, have some promising therapeutic functions on cancer immunotherapy. However, the hostile tumor microenvironment and short-term persistence *in vivo* obviously limit the effectiveness of the ACT therapy.

Memory T cells are characterized as the long-term persistence, resistance to apoptosis and high sensitiveness to little antigen challenge. The memory T cells previously include central memory T cells (Tcm) and effector memory T cells (Tem). Recently, a novel subset of T memory cells with strong stemness (Tscm) was found, comparing to the Tcm and Tem, Tscm are characterized with decades-years persistence *in vivo*, intensive self-renewing ability, consistent differentiation to Tcm, and high-resistance to apoptosis (3). Tscm cells play a significant long-term protective role from infections and cancers (4–7). The induction of Tscm may be a potential strategy for long-term effective ACT therapy.

As a member of the tumor necrosis factor receptor family, CD40 expresses in a range of immune cells, including DCs, B cells and activated CD8^+^ T cells (8). In recent years, CD40 activation can strengthen the immune responses induced by various vaccines (8, 9) and can generate long-time anti-tumor effects (10). CD40 agonist combination with chemotherapy have a higher response in various solid tumors than chemotherapy alone (11). And a tumor-targeting CD40 bispecific compound, ABBV-428, has been applied for clinical immunotherapy (12). It's reported that the activation of CD40/CD40L pathway in APCs induces the transformation of naïve CD8^+^ T cells into CD8^+^ central memory T cells (13, 14). However, the effect of CD40 in mediating the formation of Tscm, especially tumor-specific Tscm, is still unclear.

Activating CD40 signaling maximally stimulates T cell production of Wnt10b, resulting in Wnt signaling activation (15, 16). And increasing evidences reveal that Wnt/β-catenin signaling can promote CD8^+^ T cell memory formation and enhance their functionality (17, 18). Tscm cells are the least differentiated subset and possess characteristics of conventional memory T cells (3). Therefore, it's very likely that Wnt/β-catenin signaling is a critical player in the process of generating Tscm. Luca et.al. demonstrated the key role for Wnt/β-catenin signaling in the maintenance of stemness in memory CD8^+^ T cells (18).

Therefore, our laboratory discovered a possible mechanism of CD40 triggering a long-term antitumor immunity based on HPV16 specific Tscm, and developed an optimal immunization pattern for therapeutic purpose, which may provide a promising therapeutic strategy for HPV16-positive tumors.

## Materials and Methods

### Cell Lines

Mouse cervical cell line TC-1 with HPV16 E7^+^ was purchased from the American Type Culture Collection (ATCC). TC-1 cells were cultured in DMEM (Gibco) supplemented with 10% FBS (Gibco).

### Mice

Six- to eight-week-old female C57BL/6 mice and nude mice were obtained from the Experimental Animal Center at Southern Medical University (Guangzhou, PR China). Animal care and experiments were approved by the Institutional Animal Research Ethics Committee of Southern Medical University.

### Clinical Samples

Peripheral blood mononuclear cells (PBMCs) were isolated from voluntarily consenting only HPV16 positive or CSCC patients without preoperative radiotherapy or chemotherapy at the department of Gynecological Oncology of the first affiliated hospital of Guangzhou medical university (Guangzhou, China) or healthy donors between 2018 and 2019 using FicollPaque Plus (GE Healthcare). Detailed information on clinical specimens are summarized in Table S1. The study was approved by the Institutional Research Ethics Committee of Southern Medical University.

### Peptides, Antibodies, and Reagents

Optimal peptides based on HPV16 E7(RAHYNIVTF) peptides were synthesized by Chinese Peptide Ltd. The PE-labeled H-2Db/E7tetramers and HLA-A^*^0201 HPV16 E7 tetramers were purchased from MBL. The antibodies used in our study included anti-mouse CD8a (Clone:KT15, MBL), CD44(Clone:IM7, thermo fisher scientific), CD62L (Clone:MEL-14, thermo fisher scientific), IFN-γ (Clone: XMG1.2, thermo fisher scientific), EOMEs (Clone:Dan11mag, thermo fisher scientific), T-bet (Clone: eBio4B10, thermo fisher scientific) and β-catenin (Clone: 4627S, CST) and anti-human CD8a (Clone:T8, MBL, S1004, BSbio), CD62L (Clone:DREG-56, Biolegend), CD95(Clone:DX2, Biolegend), CD45RA (Clone: HI100, Biolegend) and CD122(Clone: TU27, Biolegend). The 7-AAD and Fixable Viability Dyes were purchased from thermo fisher scientific. LEAF^TM^ purified anti-mouse CD40 (Clone:1C10) was purchased from Biolegend. The vaccine adjuvant Poly (I:C) (HMW) VacciGrade™ was purchased from invivogen.

### Immunization and Establishment of Therapeutic Models

1 × 10^6^ TC-1 cells were injected subcutaneously into the right flank of C57BL/6 mice. After 7 days, tumor-bearing mice were immunized with following immunization strategy groups, selectively: PBS group, HPV16 E7 peptide group, HPV16 E7 plus 50 μg Poly (I:C) group, and HPV16 E7 plus 50 μg Poly (I:C) and 20 μg aCD40 mAb group. The mice were immunized for 3 times every 7 days via *i.v*. Tumor volume was measured every 3 days and calculated by the formula: volume = (width)^2^ x length/2. Mice were euthanized when tumor size reached 1.5 cm^3^.

### Flow Cytometry Analysis

For tetramer staining, peripheral blood cells and splenocytes were incubated with indicated antibodies plus PE-labeled H-2Db/E7 tetramer for 30 min at 4°C and 7-AAD was added to differentiate dead cells.

For Intracellular staining of IFN-γ, splenocytes were collected and incubated with TC-1 cells (1:1) in the presence of BFA for 6 h to block the secretion of IFN-γ. Then cells were collected and incubated with anti-CD8a for 30 min at 4°C. Following membrane staining, cells were fixed and permeabilized with Fixation/Permeabilization buffer (Thermo fisher scientific). Cells were then intracellularly stained with anti-IFNγ for 30 min at 4°C. The fluorescence was evaluated on the Fortessa (BD) and the data was analyzed by using FlowJo software.

For intranuclear staining of EOMEs, T-bet and β-catenin, splenocytes were collected and incubated with anti-CD8a for 30 min at 4°C. Following membrane staining, cells were fixed and permeabilized with Transcription Factor Staining buffer (Thermo fisher scientific). Cells were then intranuclear stained with anti- EOMEs, T-bet and β-catenin for 30 min at 4°C. The fluorescence was evaluated on the Fortessa (BD) and the data was analyzed by using FlowJo software.

### Adoptive Cell Transfer

The murine splenocytes were stained with anti-CD8a, CD44, CD62L plus H-2Db/HPV16E7 tetramer and were sorted on MoFlo XDP (Beckman coulter). The sorted cells of Tscm (CD8^+^Tet^+^CD44^−^CD62L^+^), Tcm (CD8^+^Tet^+^CD44^+^CD62L^+^), Tem (CD8^+^Tet^+^CD44^+^CD62L^−^) were selectively injected into nude mice radiated 2.4 Gy. 5 weeks later, 1x10^6^ TC-1 cells were transplanted into mice via *s.c*. in the right flank. Tumor growth was monitored as described previously.

### Cell Counting Kit-8 (CCK-8) Assay for Cell Viability

A CCK8 assay (Beyotime) was used to determine the effect of murine splenocytes from different groups on TC-1 cells. Briefly, TC-1 cells were seeded in 96-well plates at a concentration of 9000 cells per well and incubated with murine splenocytes at indicated E/T ratios for 48 h. At the indicated time point, 10 μL of CCK-8 assay reagent was added and the plates were incubated for another 2 h. Absorbance was detected at 450 nm. The experiments were performed with three replicate wells per sample.

### Database

Level 2 gene expression profile (level 2 data) for CESC patients was obtained from the UCSC Xena website (https://xenabrowser.net/datapages/). The gene expression profile was measured experimentally using the Illumina HiSeq 2000 RNA Sequencing and showed the gene-level transcription estimates, as in log2(x+1) transformed RSEM normalized count. Clinical data such as survival and outcome were also downloaded from UCSC Xena website. Relationship between CD40 and CD62LCD45RACD95CD122 was calculated by using an R package ggstatsplot.

### Statistical Analysis

Statistical analysis was conducted using *T*-test, and analysis between multiple groups was performed by one-way analysis of variance (ANOVA). Correlation analysis was performed using the log-rank test. Differences were considered statistically significant at *P* < 0.05.

## Results

### CD40 Enhances the Long-Term HPV-Specific Anti-Tumor Immunity *in vivo*

**Figure 1:**
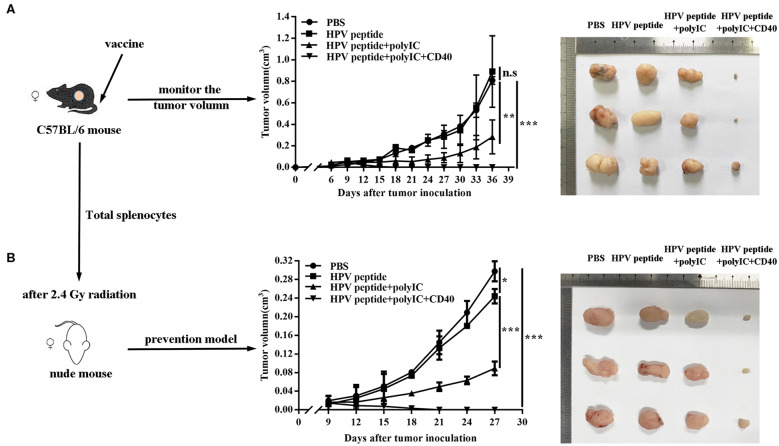
Anti-tumor effects induced by different immunization. **(A)** Mice (*n* = 3 per group) were inoculated (s.c.) with 1 × 10^6^ TC-1 cells and vaccinated (i.v.) 6 days later with PBS, HPV16 E7 peptide alone, peptide plus polyIC, peptide and polyIC plus αCD40 mAb. **(B)** In the preventive model, nude mice after 2.4 Gy radiation were injected with 10 million of total splenocytes of the immunized mice when TC-1 tumor appeared. Tumor growth was measured (two opposing diameters) and recorded once every 3 days. Data shown represent three independent experiments with similar results. *p < 0.05, **p < 0.01, ***p < 0.001, n.s, *p* > 0.05.

To study anti-tumor effects of different immunizations using a peptide epitope from HPV16 E7, we immunized HPV16^+^ tumor-bearing mice with HPV16 E7 peptide in combination with polyIC and aCD40. Comparing with controls, CD40 activation groups could obviously suppress TC-1 tumor growth (Figure 1A). To confirm the specific anti-tumor immunity, the splenocytes were harvested and adoptively transferred into nude mice, and prevented the TC-1 tumor challenge effectively (Figure 1B).

### CD40 Induces the HPV16-Specific CD8^+^ T Cell Against Cancer Cells

**Figure 2:**
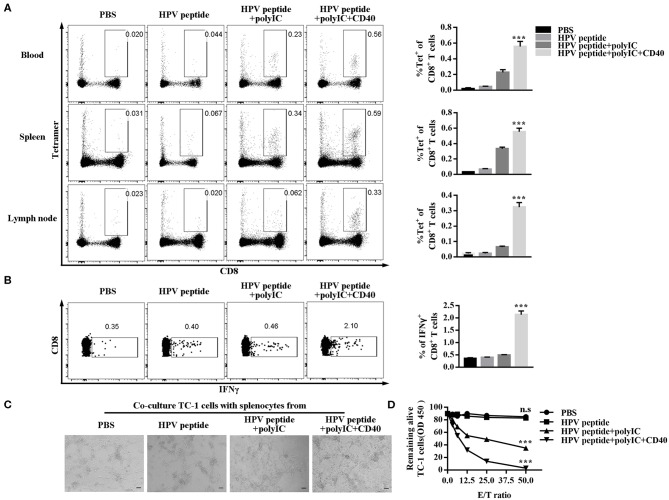
Evaluation of specific CD8^+^ T cells elicited by immunized mice and ability of killing TC-1 cells *in vitro*. **(A)** Frequencies of HPV16-specific CD8^+^ T cells in blood, spleen and lymph node from each group (3 mice/group) were measured by tetramer analysis 1 months after prime and boost induced by immunization using various vaccine formulations. **(B)** Splenocytes of the primed and boosted mice (*n* = 3 per group) were co-cultured with TC-1 cells for 6 h and analyzed for IFNγ in CD8^+^ T cells. **(C)** The cytotoxicity of the immunized mouse splenocytes was studied by co-culturing them with TC-1 cells. Pictures were taken after overnight co-culture. Scale bar = 100 μm. **(D)** CCK8 assay was then performed to determine the remaining live TC-1 cells after splenocytes were rinsed away. The CCK8 data of TC-1 tumor cells co-cultured with different ratios of splenocytes was compared to CCK8 data of TC-1 tumor cells alone to calculate % of live cells. Shown is the dose-dependent killing of TC-1 cells by splenocytes. Error bars represent the mean ± SD of three independent experiments. ***p < 0.001, n.s, *p* > 0.05.

To explore anti-tumor effect of specific CD8^+^ T cells, we detected the percentage of HPV16 E7 specific (tetramer positive) CD8^+^ T cells in mouse blood, spleen and lymph node. As shown in Figure 2A, CD40 stimulation induced more HPV16 E7 specific CD8 formation than other groups. Consistently, CD8^+^ T cells from CD40-activated mice produced more IFN-γ after co-culture with TC-1 cells (Figure 2B), and eliminated more HPV16^+^ TC-1 cells in a dose-dependent manner (Figures 2C,D).

### CD40 Facilitates HPV16-Specific CD8^+^ Tscm Formation

**Figure 3:**
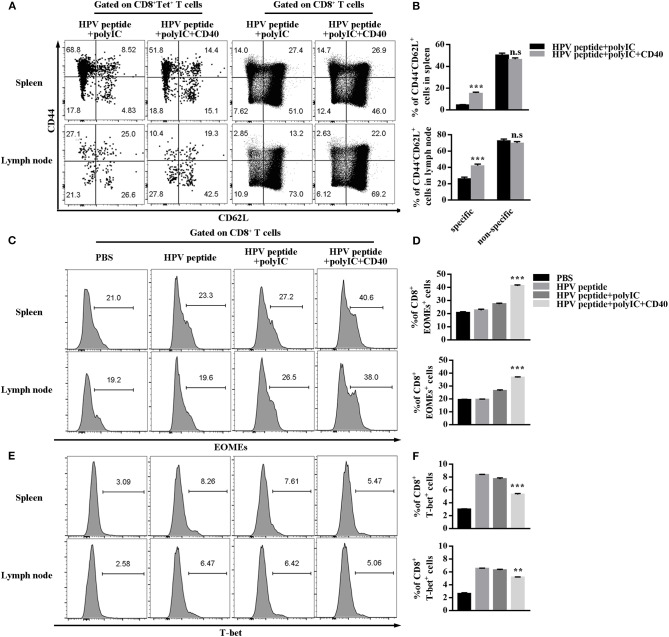
HPV16 E7 peptide in combination with polyIC and aCD40 generates more Tscm and affects the expression of EOMEs and T-bet. **(A,B)** Distributions of HPV16 E7 specific and non-specific CD8^+^ memory T cells in spleen and lymph node were measured by FCS analysis 1 months after prime and boost induced by immunization using various vaccine formulations. Frequencies of EOMEs **(C,D)** and T-bet **(E,F)** gated on CD8^+^ T cells in spleen and lymph node were measured by FCS analysis 1 months after prime and boost induced by immunization using various vaccine formulations. Error bars represent the mean ± SD of three independent experiments. **p < 0.01, ***p < 0.001, n.s, *p* > 0.05.

Long-term immune memory toward tumor antigen plays a critical role in anti-tumor effects (10). To investigate potential mechanisms underpinning CD40-amplified antitumor immunity, we analyzed the memory subsets of specific CD8 T cells by staining with CD44 and CD62L. The experiment demonstrated in Figure 3A shows CD40 stimulation induced more systemic HPV-specific CD8^+^ Tscm than other immunizations in spleen and lymph node, but not non-specific Tscm (Figures 3A,B). The transcriptional factors Eomes and T-bet balanced the differentiation and maintenance of CD8 memory T cells (19, 20). Therefore, we found that CD40 stimulation enhanced Eomes expression, but down-regulated T-bet expression (Figures 3C–F).

### Adoptive Transfer of HPV16 E7 Specific Tscm Represses Cancer Growth

**Figure 4:**
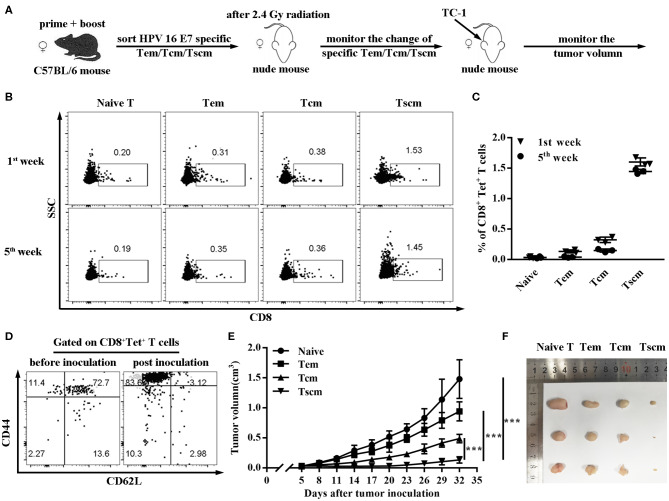
Adoptive transfer of HPV16 E7 specific Tscm represses cancer growth**. (A–C)** Frequencies of CD8^+^ T cell in blood were measured by FCS analysis 1st and 5th week after injecting different subtypes of HPV-16 specific memory T cells (Tem, Tcm, Tscm) induced by immunized C57BL/6 mice into nude mice. **(D)** After injecting different memory T cells (Tem, Tcm, Tscm) induced by immunized C57BL/6 mice into nude mice, distributions of HPV16 specific CD8^+^ memory T cells in blood were measured by FCS analysis 1st and 5th week. **(E,F)** Nude mice (*n* = 3 per group) after 2.4 Gy radiation were injected with different memory T cells of the immunized mice and were inoculated (s.c.) with 1 × 10^6^ TC-1 cells. Tumor growth was measured (two opposing diameters) and recorded once every 3 days. Data are representative of three independent experiments with similar results. ***p < 0.001.

To further verify the critical role of Tscm in preventing tumor, we sorted the subsets of HPV16 E7 specific CD8^+^ memory T cells (Tem, Tcm, Tscm) from CD40 immunized mice, and, respectively transferred different memory T cells into nude mice (Figure 4A). The data in Figure 4B shows the HPV16 specific CD8 T^+^ cells differentiation in Tscm group was significantly stronger than the groups of Tcm and Tem (Figures 4B,C), and Tscm differentiated into specific Tcm and Tem after TC-1 cells challenge (Figure 4D). Consistently, the adoptive transferring of CD8^+^ Tscm significantly repressed the tumor growth, comparing to the control groups (Figures 4E,F).

### Wnt/β-catenin Signaling Involves With CD40-Inducible HPV16-Specific CD8 Tscm Cells

**Figure 5:**
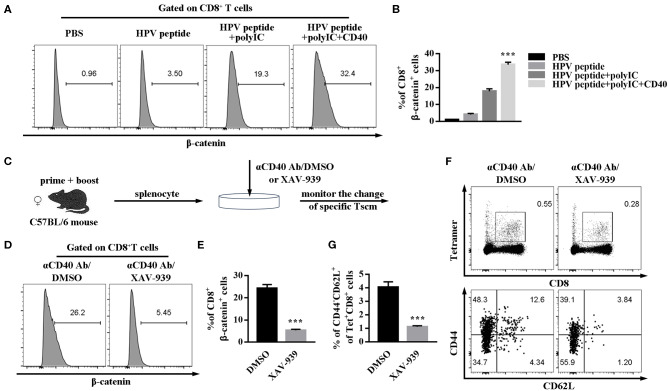
CD40 regulates Wnt/β-catenin signaling to elicit a high level of HPV16-specific CD8 Tscm cells. **(A,B)** Flow analysis of β-catenin in CD8^+^ T cells from C57BL/6 mice immunized by different vaccination strategies. **(C)** Splenocytes from immunized mice were treated with aCD40mAb plus DMSO or aCD40mAb plus XAV-939 for 48 h, then the percentage of β-catenin in CD8^+^ T cells was measured **(D,E)** and HPV16 E7 specific CD8^+^ Tscm cells **(F,G)** was detected by FCS analysis. Error bars represent the mean ± SD of three independent experiments. ***p < 0.001.

Wnt/β-catenin signaling can promote CD8^+^ T cell memory formation and enhance their functionality (17, 18). To further study the possible intrinsic mechanism of CD40 function on specific CD8 Tscm cells, the critical regulator of β-catenin in CD8 T cells was examined. CD40 stimulation was proved to upregulate β-catenin level in CD8^+^ T cells (Figures 5A,B). For making certain the role of Wnt/β-catenin signaling, β-catenin inhibitor (XAV-939) was found to block the CD40-mediated HPV16-specific CD8^+^ Tscm differentiation. The percentage of β-catenin in CD8 cells treated with aCD40mAb plus XAV-939 was much lower than aCD40mAb alone (Figures 5C–E). Consistent with results *in vivo*, it was observed that after aCD40mAb stimulation for 48 h, the proportion of HPV16 E7-specific CD8 Tscm cells came up to 4.34%, but blockade of Wnt/β-catenin by incubating with XAV-939 reversed the effect (Figures 5F,G). These data suggest that Wnt/β-catenin activation is necessary for CD40 supportive function on HPV16-specific CD8 Tscm cells.

### High Frequencies of Tscm Might Prevent the HPV Infection to Move on to the Development of Cancer

**Figure 6:**
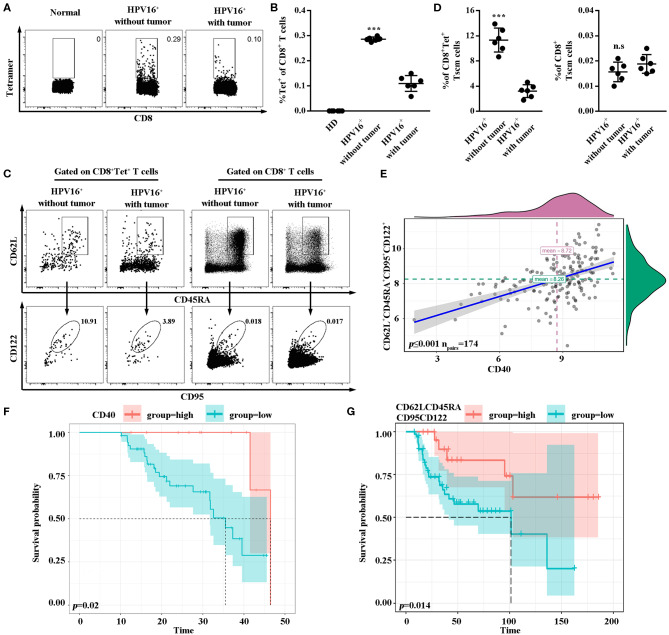
High frequencies of Tscm might prevent the HPV infection to move on to the development of cancer. **(A,B)** Flow cytometry analysis of HPV16-specific CD8^+^ T cells in PBMC from healthy donors (*n* = 6), HPV16 positive patients without tumor (*n* = 6) and HPV16 positive patients with cervical cancer (*n* = 6). **(C)** HPV16-specific or non-specific CD8 Tscm cells defined as CD45RA^+^CD62L^+^CD95^+^CD122^+^ from CD8^+^ or CD8^+^Tet^+^T cells in PBMC from HPV16 positive patients without or with cervical cancer were examined by flow cytometry. **(D)** The frequency of specific and non-specific CD8 Tscm cells in PBMC from HPV16 positive patients without tumor (*n* = 6) and HPV16 positive patients with cervical cancer (*n* = 6). **(E)** Relationship between CD40 and CD62L^+^CD45RA^+^CD95^+^CD122^+^ were shown (n_pairs_ = 174). **(F,G)** K-M plots of HPV16 positive patients with cervical cancer for CD40 **(F)** and CD62LCD45RACD95CD122 **(G)** respectively. Error bars represent the mean ± SD of three independent experiments. ***p < 0.001; n.s, *p* > 0.05.

Lastly, to confirm the association between our hypothesis and clinical prognosis, HPV16-specific Tscm cells marked by tetramer staining were examined in PBMC of healthy female donors (*n* = 6), HPV16 infected patients (*n* = 6) and HPV16 positive cervical cancer patients (*n* = 6). As shown in Figures 6A,B, the level of HPV16-specific CD8 T cells was higher in patients with HPV16 without tumor, comparing to the cancer patients. Moreover, the level of human HPV16-specific CD8^+^ Tscm (CD8^+^tet^+^CD45RA^+^CD62L^+^CD95^+^CD122^+^) in HPV16 infected patients without tumor, was higher than that of cancer patients (Figures 6C,D), indicating that HPV16-specific Tscm cells might prevent the HPV infection to move on to the development of cervical cancer. Correlation analysis demonstrated CD40 protein was positively associated with stem cell marker CD62L^+^CD45RA^+^CD95^+^CD122^+^ (Figure 6E). Kaplan-Meier survival estimates, based on dichotomized protein expression data, subsequently confirmed that patients with cervical cancer with high CD40 and high CD62LCD45RACD95CD122 protein expression had significantly longer OS (CD40, *p* < 0.05; CD62LCD45RACD95CD122, *p* < 0.05) (Figures 6F,G).

## Discussion

Adoptive T-cell immunotherapy is a promising approach to cancer therapy, especially TCR or CAR gene engineered effector T cells. However, there are functional challenges of engineered T cells therapy, including short-persistence of T cells in the tumor microenvironment (21, 22). Therefore, the long-lived T cell is an urgent need for cancer immunotherapy. Our findings demonstrated that effective induction of antigen-specific CD8^+^ Tscm with CD40 activation evoked long-term anti-tumor immunity. Firstly, CD40 stimulation induced long-lived HPV-specific CD8^+^ Tscm, which consistently differentiated into Tcm and Tem, and evoked persistent anti-tumor response. Secondly, CD40 stimulation activated Wnt/β-catenin to reprogramme stem-like characteristic of CD8^+^ T cells. Lastly, the clinical evidence confirmed that CD40 induced HPV16-specific Tscm cells need to be enhanced in cancer patients to prevent further progression of the disease or to induce even cure, and may be adopted as a potent immunotherapy.

As novel memory T cells, Tscm cells obviously have enhanced characteristics of the conventional memory T cells. Comparing to the Tcm and Tem, Tscm could be recalled with little antigen stimulation threshold (3). Tscm could be effective surveillance on little cancer cells appearance, and eliminate them at the early phase. Our data suggested that Tscm were very different from other memory T cell subsets in anti-tumor effects, including Tem and Tcm. Compared with Tem and Tcm, Tscm have a lower ratio but a greater life span and superior anti-tumor response (23). Furthermore, the multiple hostile components of cancer cells and stromal cells in tumor microenvironment, such as immune checkpoints, hypoxia, amino acid deprivation, block anti-tumor T cells function and survival. The superior ability of self-renewing and apoptosis-resistance contributes to Tscm survival in tumor. Expression of CD95 by long-lived memory T cells promotes cell proliferation by inducing TCR-β-catenin signaling, which is important for their self-renewal and persistence (24). The levels of anti-apoptotic genes such as *BCL2* and *MCL1* are specifically upregulated in Tscm cells, which accounts for their enhanced survival capacity when little or no antigen exists (25). More importantly, multidimensional scaling analysis demonstrated that Tscm cells were the most similar to naïve T cells because there were only 75 different genes between them compared to 157 and 226 for Tcm and Tem cells (3), thus which could continuously differentiate into Tcm, Tem and CTL under antigen stimulation. Because of the potent ability of Tscm, the Tscm induction becomes an important indicator for estimating various vaccines because of its special characteristics, such as the yellow fever vaccine YF-17D (26) and the AIDS experimental vaccine RV144(27). This may bring a new hope to solve existing problems in current rising T cell adoptive immunotherapy, where TCR or CAR gene engineered effector T cells easily become exhaustion and are hard to survive for a long time (28). Clinical trials have demonstrated that only 1700 Tscm clones persisted and maintained their properties for up to 12 years in patients with genetically modified T memory stem cells (29). The engineered Tscm cells have been proved to be safe, functional and decade-long survival, which is particularly attractive to be used for adoptive immunotherapies (23).

In our previous study, we proved that Tscm induced by the immunization with lv-expressing epitope-optimized antigen peptides priming and peptide boosting could undergo more *in vivo* expansion than Tcm and Teff upon re-encountering antigen and had a long-lasting anti-tumor effect (7). Recently, many groups have reported that Wnt or Notch signaling induced-Tscm cells could provide breakthrough in developing target-specific T cell therapy (3). Here, we proposed a novel strategy of generating peptide vaccine-induced Tscm cells and certified that the adoptive transfer of low number of T cells from Tscm subset can long term maintain immune responses and effectively prevent tumor recurrence *in vivo*. A number of clinical trials about peptide vaccines treating tumors have been developed for many years. Most of studies concentrate on melanomas because of its systemic tolerance toward conventional therapies and high immunogenicity. Obviously, peptide vaccines possess extremely high security, easy synthesis and purification and induce specific immune response comparing with other immunotherapies while they may be easy to degrade within a short time and acquire short-lived effects. Here, we attempt to improve current therapy toward cervical cancer, especially its recurrence, according to using CD40-based HPV16 E7 vaccine which solves problems existing in peptide vaccines. Moreover, our strategy promotes long-term protection of vaccine comparing with HPV vaccine currently on the market. The combinatorial use of peptide vaccines with CD40 antibody and TLR3 agonists acquires not only greater therapeutic efficacy but also positive preventive effect. Our method has an advantage in generating Tscm cells compared with Wnt (18) or Notch signaling activators (30) since they need to convert memory T cells into Tscm cells but we can directly gain antigen-specific Tscm cells. In our study, it's worth noting that CD40 vaccine can only inhibit larger tumor (>1 cm^3^) for several days but can completely eliminate smaller tumor (<0.2 cm^3^), what's more, CD40 vaccine can suppress the growth of tumor in early stage until it disappears. The meta-analysis identified 14 randomized clinical trials that the early or late use of targeted drugs has no effect on the survival curve of patients with non-small cell lung cancer, but the overall risk of death in patients who take the first use of immune drugs is more than 30% lower than that of patients who take immune drugs after getting other therapies is also in agreement with our data (31). These data indicates CD40-based peptide vaccines are suitable to patients with less tumor burden or greater risk of recurrence. Of course, severe systemic toxicity of CD40 antibody, including elevated liver enzymes and cytokine release syndrome, should be noticeable in clinical studies. However, Fransen et.al. found that local delivery and slow release of agonistic anti-CD40 Ab to the tumor-draining area can effectively activate local tumor-specific CD8 T cells to become systemic effectors without systemic toxicity or non-specific CTL activation (32). And recent studies demonstrate that it's possible to engineer a tumor-targeted CD40 molecule by conjugating a CD40 agonist mAb chemically with a tumor-homing peptide could potentially maximize antitumor potency while limiting systemic toxicity in clinical studies (33, 34). Therefore, it's necessary to explore the effect of these routes on HPV-specific Tscm cells and their safety in clinical studies.

A more interesting thing was that the ratio of non-specific Tscm cells in peripheral blood was much less than antigen-specific Tscm cells whether the patients developed cancer or not. These results indicated that Tscm cells possessed stronger antigen specificity during pathogens infection. But Hong et al. showed that the frequency of Tscm cells in NSCLC patients obviously distinct from the healthy donors, including in the blood, lymph nodes and TILs (35, 36). They analyzed the characteristics of T cell subsets in NSCLC patients which didn't take tumor specificity into account. Perhaps most of immune cells surrounded by tumor cells in TIL have been educated into antigen-specific cells. Besides, it is uncertain whether the different sorts of cancers may cause the different proportion of Tscm cells. Because Speiser and his colleagues demonstrated that yellow-fever specific Tscm could provide a long-term protection for humans (6). Antiviral responses largely correlated to the change of human CD8^+^ T cell subsets (6). And recent study has demonstrated that tumor cells prevented stem-like CD8 T cells from forming immune niches within tumors in order to escape anti-tumor immune response, which explains why there was fewer CD8 Tscm cells in intra-tumoral niche. This finding is consistent with our data acquired from blood, spleen and lymph node (37).

Multiple studies have proved that CD40 plays a unique role in motivating adaptive immunity. Activated CD40 signaling facilitates long-lived memory CD8 T cells differentiation while CD8^+^ T cells can't differentiate into memory cells without CD40 protein (38, 39). CD40 has been considered to promote the generation of CD8^+^ central memory T cells (14). APCs infected by CD40L-expressing recombinant vaccinia virus promote the differentiation of naïve CD8^+^ T cell (CD8^+^CD45RO^−^CD62L^+^) into central memory-like cells (CD8^+^CD45RO^+^CD62L^+^)(14). However, little is known about the role of CD40 in Tscm. In our study, we observed that the immunization of combined polyIC and CD40 were capable of inducing expansive HPV16 E7-specific CD8 Tscm cells more significantly than Tcm subset. And obviously, the activation of CD40 signaling immensely facilitates the generation of specific Tscm cells rather than non-specific Tscm cells. There have been few reports on antigen-specific Tscm cells because low frequency of these cells limits detailed characterization (23). But it's evident that only antigen-specific Tscm can elicit long-lasting cellular immune response to tumor antigen (23). Checkpoint inhibitors offer clinical benefit to a limited number of patients, and response is heterogeneous, mainly because there are different levels of immunogenicity among cancer subtypes. And, treatment with these drugs is also often associated with the induction of adverse autoimmune reactions due to the lack of specificity of therapeutic effects (40). That's why engineering T-cells with chimeric antigen receptors (CARs) and TCRs that target specific tumor antigens and re-infusing them into patients have produced encouraging clinical outcomes in clinical trials (41, 42). Our data is very meaningful for solving off-target effect and short-time response in current immunotherapy. Thus, understanding mechanisms how CD40 contributes to the generation of CD8 Tscm cells may develop addition immune strategies for therapeutic vaccines.

Wnt/β-catenin signaling has emerged as a key regulator in T cell development, including cell survival and stemness maintenance (43, 44). Constitutive Wnt signaling activation favors the formation of more antigen- specific memory CD8 T cells during initial immunization (17). Memory CD8 T cells with high Wnt signaling display increase recall proliferation capacity compared with memory cells with low Wnt level, such as short-lived effector cells and effector-like memory cells (45). More recently, a number of evidences reveal that Wnt/β-catenin signaling promotes the generation of CD8 memory stem cells and maintains stemness in CD8 memory cells according to arresting effector T cell differentiation (18). This is consistent with our results that Wnt signaling inhibitor XAV-939 could downregulate the level of CD8 Tscm cells. CD40L, which sustains T cell activation by binding to CD40 (16, 46), has been regarded as the upstream of Wnt10b primarily produced by activated T cells (47). In WT mice, Wnt signaling can be activated and Wnt10b increasingly produces by T cell but not in CD40^−/−^ mice (**?**). The notion that Wnt10b is primarily produced by activated T cells (47) and CD40L is a T cell surface receptor required for sustaining T cell activation (48) accounts for the finding. Collectively, these findings indicate the critical role of CD40 in Wnt signaling activation. However, whether CD40 potentiates antigen-specific CD8 Tscm generation through activating Wnt/β-catenin signaling remains unclear. Our results firstly demonstrated that CD40 promotes HPV16 E7-specific Tscm upregulation in a Wnt-dependent manner. Recent studies indicate that Tscm can accumulate by activating Wnt signaling *in vitro* in PBMC from RCC patients (49), which explains why CD40 can increase the proportion of antigen-specific Tscm and may help to develop Tscm-based adoptive immunotherapy.

In summary, our data demonstrates CD40 potentiates the antigen-specific CD8^+^ Tscm cells through Wnt/β-catenin signaling to facilitate the effective long-term CD8 T cell based immunotherapy, and could develop an optimal immunization strategy against cervical cancer, and these HPV16-specific Tscm cells are associated with progression risks in CSCC patients. Our study may provide a novel diagnosis and therapy strategy for immunotherapy.

## Data Availability Statement

The raw data supporting the conclusions of this article will be made available by the authors, without undue reservation, to any qualified researcher.

## Ethics Statement

The studies involving human participants were reviewed and approved by the Institutional Research Ethics Committee of Southern Medical University. The patients/participants provided their written informed consent to participate in this study. The animal study was reviewed and approved by the Institutional Research Ethics Committee of Southern Medical University.

## Author Contributions

SW, WW and YZ designed research. YZ, NW, MD, YY, ZW and RY performed experiments. YZ, NW, LH, WZ, AM, XH, CZ and SW analyzed data. YZ and SW wrote the manuscript.

## Conflict of Interest

The authors declare that the research was conducted in the absence of any commercial or financial relationships that could be construed as a potential conflict of interest.
