# Supplementary material for: CD40 Accelerates the Antigen-Specific Stem-Like Memory CD8+ T Cells Formation and Human Papilloma Virus (HPV)-Positive Tumor Eradication
Source: Front Immunol. 2020 May 27;11:1012. doi: 10.3389/fimmu.2020.01012 (PMC7267052; doi:10.3389/fimmu.2020.01012)
Supplement: Supplementary file 1 [file Table_1.DOCX]

| Patient | Age | HLA type | HPV type | Histology | Stage |
| --- | --- | --- | --- | --- | --- |
| 1 | 42 | 02 | no | healthy | no |
| 2 | 40 | 02 | no | healthy | no |
| 3 | 45 | 02 | no | healthy | no |
| 4 | 39 | 02 | no | healthy | no |
| 5 | 52 | 02 | no | healthy | no |
| 6 | 50 | 02 | no | healthy | no |
| 7 | 52 | 02 | 16 | CIN | 1 |
| 8 | 47 | 02 | 16 | CIN | 2 |
| 9 | 43 | 02 | 16 | CIN | 1 |
| 10 | 39 | 02 | 16 | CIN | 1 |
| 11 | 46 | 02 | 16 | CIN | 2 |
| 12 | 40 | 02 | 16 | CIN | 1 |
| 13 | 51 | 02 | 16 | Squamous | IB1 |
| 14 | 55 | 02 | 16 | Squamous | IB1 |
| 15 | 50 | 02 | 16 | Squamous | IB2 |
| 16 | 45 | 02 | 16 | Squamous | IB1 |
| 17 | 35 | 02 | 16 | Squamous | IIB |
| 18 | 42 | 02 | 16 | Squamous | IIA1 |

**Supplementary Tables**

**Table S1. Clinical characteristics of patients in the study, related to Fig 6.**
